# Perceived barriers in family‐based behavioural treatment of paediatric obesity – Results from the FABO study

**DOI:** 10.1111/ijpo.12992

**Published:** 2022-12-05

**Authors:** Hanna F. Skjåkødegård, Sigurd Hystad, Ingvild Bruserud, Rachel P. K. Conlon, Denise Wilfley, Bente Frisk, Mathieu Roelants, Petur B. Juliusson, Yngvild S. Danielsen

**Affiliations:** ^1^ Department of Clinical Science University of Bergen Bergen Norway; ^2^ Department of Psychosocial Science University of Bergen Bergen Norway; ^3^ Faculty of Health VID Specialized University Bergen Norway; ^4^ Department of Pediatrics Haukeland University Hospital Bergen Norway; ^5^ Department of Psychiatry University of Pittsburgh School of Medicine Pittsburgh Pennsylvania USA; ^6^ Department of Psychiatry Washington University School of Medicine St. Louis Missouri USA; ^7^ Department of Health and Functioning Western Norway University of Applied Sciences Bergen Norway; ^8^ Department of Physiotherapy Haukeland University Hospital Bergen Norway; ^9^ Department of Public Health and Primary Care KU Leuven, University of Leuven Leuven Belgium; ^10^ Children and Youth Clinic Haukeland University Hospital Bergen Norway; ^11^ Department of Health Registry Research and Development Norwegian Institute of Public Health Bergen Norway; ^12^ Department of Clinical Psychology University of Bergen Bergen Norway

**Keywords:** adolescent, attrition, barriers to treatment, children, dropout, family‐based treatment, paediatric obesity

## Abstract

**Background:**

To date, few studies have investigated perceived barriers among those who participate in and drop out of family‐based behavioural treatment (FBT) for paediatric obesity. Examining experienced barriers during treatment, and their role in participation and completion of treatment has important implications for clinical practice.

**Objectives:**

To compare perceived barriers to participating in a family‐based behavioural social facilitation treatment (FBSFT) for obesity among families who completed and did not complete treatment.

**Methods:**

Data were analysed from 90 families of children and adolescents (mean (*M)* age = 12.8 years, standard deviation (*SD)* = 3.05) with severe obesity enrolled in a 17‐session FBSFT program. After completing 12 sessions or at the time of dropout, parents and therapists completed the *Barriers to Treatment Participation Scale* (BTPS), a 5‐point Likert scale (1 = never a problem, 5 = very often a problem) which includes four subscales: 1. *Stressors and obstacles that compete with treatment*, 2. *Treatment demands and issues*, 3. *Perceived relevance of treatment*, 4. *Relationship with the therapist*.

**Results:**

Families who did not complete treatment scored significantly higher on the BTPS subscales *stressors and obstacles that compete with treatment* (*M* = 2.03, *SD* = 0.53 vs. *M* = 1.70, *SD* = 0.42), *p* = 0.010 and *perceived relevance of treatment* (*M* = 2.27, *SD* = 0.48 vs. *M* = 1.80, *SD* = 0.50), *p* < 0.001 than families who completed treatment. No other significant differences between groups were observed.

**Conclusion:**

Families are more likely to drop out of FBSFT when experiencing a high burden from life stressors or when treatment is not meeting the expectations and perceived needs of the family.

**Abbreviations**
BTPS: barriers to treatment participation scale
FBT: family‐based behavioural treatment
FABO: family‐based behavioural treatment of childhood obesity study
RCT: randomized controlled study
FBSFT: family‐based behavioural social facilitation treatment
IOTF: international obesity task force
BMI: body mass index
SDS: standard deviation score
SD: standard deviation

## 1 INTRODUCTION

Paediatric obesity, recognized as a global health challenge for decades, is now further exacerbated in the Covid‐19 pandemic.^1^ In this context, efforts to develop effective interventions for children with obesity are critically important, especially addressing the high risk of attrition from intervention programming that impairs disease control and decreases treatment effectiveness.^2^, ^3^, ^4^, ^5^, ^6^ Examining the barriers families experience during treatment, and the role these barriers play in participation and completion of treatment, offers an opportunity to improve delivery methods, identify families at risk for dropping out, and tailoring the treatment to improve compliance and impact.

The majority of studies on attrition from paediatric obesity treatment have focused on pre‐treatment predictors,^2^, ^7^ commonly comprised of demographic variables, for example, age, sex, initial body weight and socioeconomic status.^7^ Previous dieting attempts, psychopathology and body image have also been investigated, all with mixed findings regarding their ability to predict attrition.^2^ The lack of consistent findings can result from differences in the target populations, treatment approaches and definitions of attrition between studies.^2^, ^7^ Nevertheless, the inconsistent findings indicate that factors other than pre‐treatment predictors may play an important role for treatment retention.^2^ Efforts to identify these factors, and thereby make it possible to develop strategies to enhance retention rates and prevent dropout are highly needed.^5^ To address this, the *Barriers to Treatment Participation Scale* (BTPS)^8^, ^9^ has been proposed as a suitable measure to identify factors perceived as barriers for participation in paediatric obesity treatment.^7^

To date, few studies have investigated perceived barriers for treatment participation in lifestyle interventions for paediatric obesity.^2^, ^6^, ^10^ The existing studies, are mainly qualitative, and report that a high burden from life stressors (e.g., single parent household with multiple children, parental chronic illnesses, limited means and logistical challenges) forms a complex interplay of barriers interfering with treatment participation.^2^, ^6^, ^11^, ^12^, ^13^ Interestingly, logistical challenges have been put forward as more related to treatment attrition than program satisfaction.^4^, ^12^, ^14^, ^15^ It seems like busy work schedules for parents, lack of transportation and insurance coverage may contribute to attrition despite low degree of dissatisfaction with the programs.^4^, ^14^, ^16^ Furthermore, it is worth noting, that previous research on barriers for participating in lifestyle treatment for paediatric obesity has mainly focused on those who did not complete treatment, without comparison of experienced barriers among those who completed treatment,^2^, ^6^ resulting in a lack of knowledge related to similarities and differences in reported barriers between the two groups.^2^

Family‐based behavioural treatment (FBT) is an evidence‐based intervention for paediatric obesity, shown to yield clinically significant weight loss.^17^, ^18^, ^19^ Investigating barriers for participation in this kind of treatment and the associations to attrition or retention is an important addition to research on pre‐ to post‐treatment change in weight and behavioural outcomes.^14^, ^19^ Studies have indicated that, in addition to family stressors, different aspects of the treatment (demands and relevance) and alliance with the therapist are likely to influence treatment attendance and outcome in psychological treatment of children and families.^8^, ^20^ These kinds of within‐treatment barriers have rarely been examined in relation to FBT for paediatric obesity. The family‐based behavioural treatment of childhood obesity (FABO) study,^21^ offers an opportunity to investigate barriers evident during and in relation to participation in an enhanced FBT for paediatric obesity.

Thus, the aim of the present study was to compare perceived barriers to treatment participation in family‐based behavioural social facilitation treatment (FBSFT) for paediatric obesity among families who did or did not complete the intervention. We hypothesized that there would be a higher level of perceived barriers among families who did not complete the treatment.

## 2 METHODS

### 2.1 Study design

**FIGURE 1:**
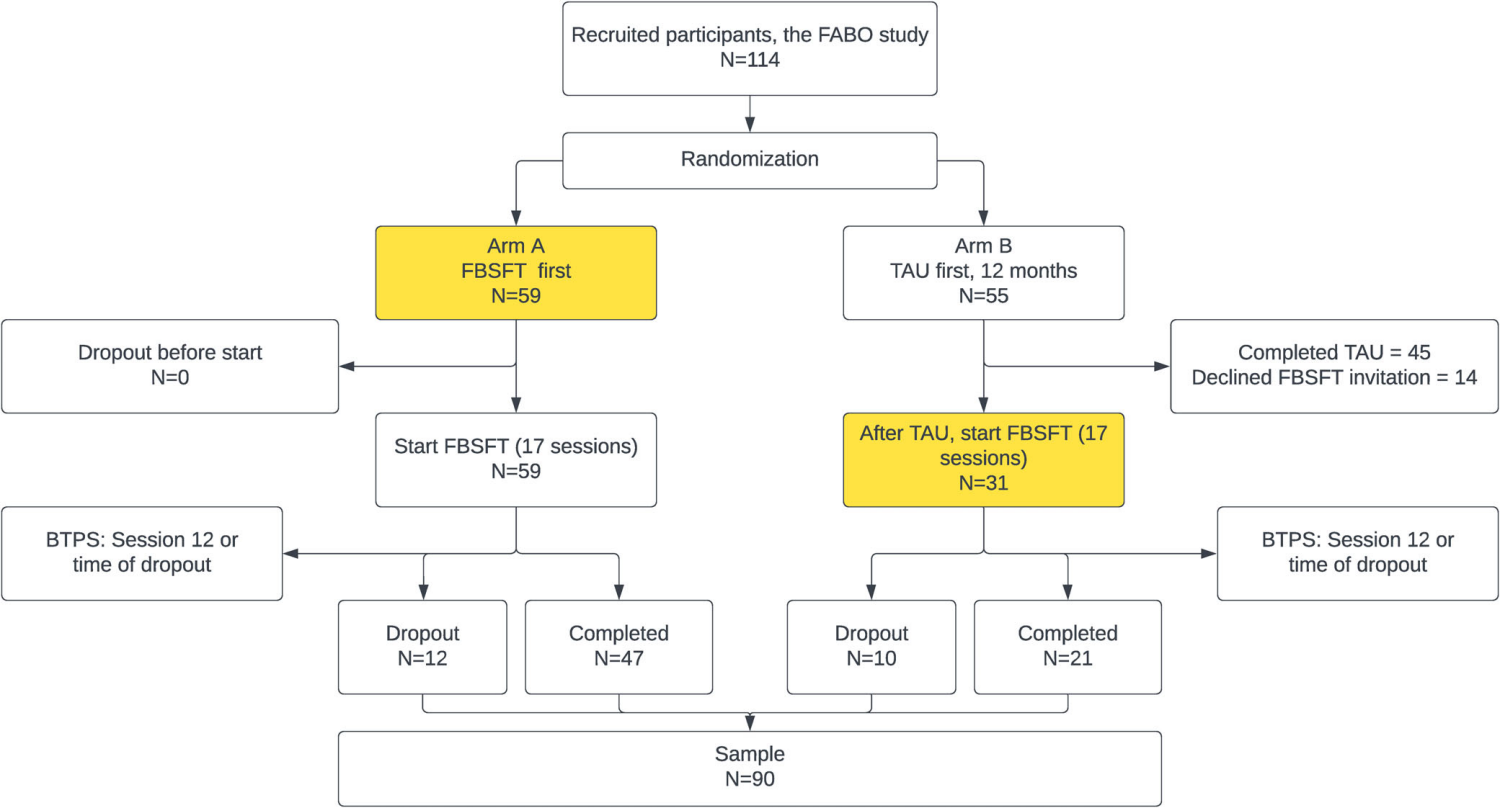
Flow chart showing the participant flow for the FBSFT‐part of the FABO study. Coloured boxes represent the baseline time points for participants included in the current study. BTPS, barriers to treatment participation scale; FBSFT, family‐based behavioural social facilitation treatment; TAU, treatment as usual

This research is part of the FABO study,^21^ a randomized controlled trial (RCT) evaluating the effect of FBSFT compared to the standard treatment given to children with severe obesity at the Obesity Outpatient Clinic, Haukeland University Hospital, Bergen, Norway.^19^, ^21^ Participants were recruited from February 2014 to October 2018. The FABO study involved a waitlist control design in which all participants eventually were offered FBSFT, and the current analysis includes data from families while participating in the FBSFT portion of the trial. Figure 1 describes the study design and participant flow. Written informed consent was obtained prior to inclusion. The consent was obtained from all participating adolescents older than 16 years, or otherwise from their parents, complemented with an informed consent when the child was 12 years of age or older.

The study was approved by the Regional Committee for Medical and Health Research Ethics, Western Norway (number 2013/1300) and was registered on ClinicalTrials.gov (NCT02687516).

### 2.2 Participants

A total of 90 families with children and adolescents (aged 6–18 years) with severe obesity are included in this analysis. Criteria for admission to the study was an International Obesity Task Force (IOTF)^22^ body mass index (BMI) ≥35 or BMI ≥30 with obesity related co‐morbidity. The child participated in the treatment together with her/his family, such that both the child and at least one of the parents agreed to actively participate. Families were excluded if either the child or parent(s) experienced severe somatic or psychiatric illness that could interfere with the treatment program, or current participation in other obesity treatment programs.

### 2.3 Description of treatment

FBSFT builds on FBT,^19^, ^21^ and consisted of 17 individual family sessions. The intention was to deliver the sessions weekly,^21^ but due to logistical challenges when delivering the treatment in a real‐world health care clinic, the treatment ended up being delivered in an unstructured combination of weekly and fortnightly sessions. Mean treatment delivery was approximately 6 months. In the sessions each family worked on changing lifestyle behaviours using a structured cognitive behavioural approach.^21^

The treatment targets healthy lifestyle changes in both children and parents in the domains of diet, physical activity, sedentary activity, sleep, and social function. Through the treatment sessions, the families are taught a set of behavioural and cognitive techniques for promoting healthy behaviour change and dealing with factors that maintain unhealthy lifestyle behaviours. There are session‐specific components and goals, and from session‐to‐session the families are encouraged to self‐monitor their behaviours and support for health behaviours in their home, peer, and community environments. Further description of the treatment is provided in the published study protocol.^21^

#### 2.3.1 Completion of treatment

Completion of treatment is defined as attending >75%^23^ (i.e., ≥13) of the 17 sessions. Families who attended <13 sessions were considered to have dropped out (i.e., did not complete treatment).

### 2.4 Anthropometric measures

Height and weight were measured by trained personnel at the Obesity Outpatient Clinic. Height was measured with a digital wall‐mounted stadiometer (Seca 264, Seca, Hamburg, Germany) and recorded to the nearest 0.1 cm. The participant was wearing underwear (without socks and shoes). Body weight was measured with a digital scale (InBody720, Biospace, Seoul, Korea) and recorded to the nearest 0.1 kg. BMI was calculated by dividing the persons weight in kilograms by the square of height in meters (kg/m^2^), and further converted to BMI standard deviation score (SDS) derived from the Norwegian growth reference.^24^

### 2.5 Demographic information

Family structure, parental education levels and parental employment were measured with a parental questionnaire at baseline.^19^ The questionnaire was part of the baseline assessment at the Obesity Outpatient Clinic.

### 2.6 Barriers for treatment measure

Barriers for treatment were investigated with the Barriers to Treatment Participation Scale (BTPS).^8^, ^9^ The BTPS was developed and validated to address dropout from treatment with outpatient psychological treatment of children and adolescents.^8^, ^9^ The main section of the questionnaire consists of 44 statements evaluated on a 5‐point Likert scale (1 = never a problem, 5 = very often a problem). Scores were distributed across four subscales: (1) *Stressors and obstacles that compete with treatment*, (2) *Treatment demands and issues*, (3) *Perceived relevance of treatment*, (4) *Relationship with the therapist*. Statements 9 and 10, related to treatment costs, and statement 37 *the therapist did not call often enough* were not applicable for our study, and were therefore excluded when calculating scores. Subscale scores are calculated using the average of the items. In addition to the four subscales, BTPS includes 14 questions about specific critical life events that are answered in a yes or no format. The purpose of these 14 questions is to distinguish perceived barriers associated with treatment participation from specific life‐changing events.^25^ The question *my medical insurance did not cover this treatment* was not applicable since the treatment was free of charge and excluded when calculating critical event score. Parent and therapist versions of BTPS were used and completed by both families and therapists either at program dropout or after completion of 12 out of the 17 FBSFT sessions. On average, session 12 was delivered in week 18 of the FBSFT program. The BTPS^8^ is outlined in Table 1 with permission from the authors. The BTPS has been found to yield high levels of internal consistency and to be predictive of treatment drop‐out, cancellation of appointments and weeks spent in treatment.^8^

**TABLE 1:** Subscales and Items of the Barriers to Treatment Participation Scale

*I* . *Stressors and obstacles that compete with treatment (20 items, Scored 1–5)*
2. Transportation (getting a ride, driving, taking a bus) to the clinic for a session
3. My child was in other activities (sport, music lessons) that made it hard to come to a session
4. Scheduling of appointment times for treatment
6. Treatment was in conflict with another of my activities (classes, job, friends)
14. During the course of treatment I experienced a lot of stress in my life
16. I was sick on the day when treatment was scheduled
17. My child was sick on the day when treatment was scheduled
18. Crises at home made it hard for me to get to a session
20. Treatment added another stressor to my life
31. There was bad weather and this made coming to treatment a problem
34. I did not have time for the assigned work
35. My child was never home to do the assigned homework
36. There was always someone sick in my home
38. Getting a baby‐sitter so I could come to the sessions
39. Finding a place to park at the clinic
40. I had a disagreement with my husband, boyfriend, or partner about whether we should come to treatment at all
41. I was too tired after work to come to a session
42. My job got in the way of coming to a session
43. Treatment took time away from spending time with my children
44. I had trouble with other children at home which made it hard to come to treatment
*II. Treatment demands and issues (10 items, Scored 1–5)*
1. My child refused to come to the session
5. Treatment lasted to long (too many weeks)
9. I felt that treatment cost too much
10. I was billed for the wrong amount
12. Information in the session and handouts seemed confusing
13. My child had trouble understanding treatment
22. I felt this treatment was more work than expected
23. The atmosphere at the clinic makes it uncomfortable for appointments
24. I did not feel that I had enough to say about what goes on in treatment
33. The assigned work for me to do as part of this treatment was much too difficult
*III. Perceived relevance of treatment (8 items, Scored 1–5)*
7. Treatment did not seem necessary
11. Treatment was not what I expected
15. I lost interest in coming to sessions
21. I felt treatment did not seem as important as the sessions continued
25. I feel treatment did not focus on my life and problems
28. My child now has new or different problems
29. My child's behaviour seems to have improved, therefore, treatment no longer seems necessary
30. Treatment did not seem to be working
*IV. Relationship with the therapist (6 items, Scored 1–5)*
8. I did not like the therapist
19. I felt I had to give too much personal information to the therapist
26. The therapist did not seem confident that treatment would work for my child
27. The therapist did not seem confident in my ability to carry out programs
32. I do not feel the therapist supported me or my efforts
37. The therapist did not call often enough
*V. Critical events (14 items, Scored yes, no)*
45. I moved to another house or apartment during the time my child was in treatment
46. My medical insurance did not cover this treatment
47. I moved to far way from clinic to come to treatments sessions (out of the area)
48. My family changed in size (another baby or someone moved in or out of the home)
49. I lost my job or had a change in income
50. I got a job or changed jobs
51. There was an alcohol or drug problem in my family
52. There was physical or sexual abuse in my family
53. A close friend or relative got very sick or died during treatment
54. My child moved out of the home
55. My child was put into an in‐patient program or residential program
57. My child changed schools during treatment
56. I had legal problems (arrest, driving violations, etc.)
58. I got separated or divorced

*Note*: Reproduced from Kazdin et al. *J Child Psychol Psychiatry*. 1997,^8^ with permission. The items constitute the parent version of the scales, the items are the same for the therapist version, with adjusted wording to convey that parent and child are to be evaluated.

### 2.7 Statistical analyses

Data were analysed with IBM SPSS version 27 (IBM Corp., Armonk, NY). Descriptive statistics of continuous variables are given by the mean and SD, and of categorical variables by the frequency and percentage. Demographic variables for the groups of families completing and not completing treatment were compared with *t*‐ and chi‐square tests for continuous and categorical variables.

To compare perceived barriers to treatment between families who did and did not complete treatment, we first calculated the four different BTPS subscales. Higher scores indicate greater presence of problems and barriers to treatment. A Hotelling's *T* ^2^ test was then used to compare the multivariate data (i.e., the BTPS subscales) between groups. A Box M test was used to test the assumption of homogeneity, that is, that both populations have a common variance–covariance matrix. Statistically significant *T* ^2^ values were followed‐up with post‐hoc comparisons of individual subscales, using independent‐samples *t*‐tests with a Bonferroni correction.

The subscale *Relationship with the therapist* was not included in the above multivariate analysis. As more than 50% of the sample had a mean score equal to 1 on this subscale, the variable was highly skewed with limited variance, and a comparison of groups was therefore not feasible or meaningful. Instead, we performed a Wilcoxon rank‐sum test to test the hypothesis that the comparison groups are from populations with the same distribution and computed the probability that a random case from one group has a higher score on *Relationship with the therapist* than a random case from the other group.

To compare critical life events between families who did and did not complete treatment, we first summed the life events questions into a composite score, and then performed a Wilcoxon rank‐sum test as explained above.

## 3 RESULTS

Baseline characteristics of the study population are presented in Table 2, both in total and for families who completed and did not complete treatment separately. No significant differences between groups were observed. Of the 90 participants (mean age 12.8 years; minimum – maximum: 5–9 to 17.7 years) who participated in the FBSFT‐part of the FABO study, 68 (75.5%) families completed treatment, while 22 (24.5%) families did not complete treatment. Mean dropout session was session 6, with session 12 representing latest dropout point.

**TABLE 2:** Characteristics of the study population at baseline, in total and by groups of families who did and did not complete treatment

		Completed	Not completed	*p* value
Total (*N*)	90	68	22	
Age (mean, SD)	12.79 (3.05)	12.7 (3.1)	13.2(2.9)	0.490
Range	5.9–17.7	5.9–17.4	10.7–17.7	
Sex: girls (%)	53 (58.9%)	42 (61.8%)	11 (50%)	0.468
BMI (mean, SD)	32.18 (4.88)	31.6(4.59)	33.9(5.46)	0.056
BMI z‐score mean (SD)	2.99 (0.49)	2.93(0.48)	3.16(0.49)	0.062
Parent reported data				
Mother born in Norway (%)	87.6%	85.3%	95.2%	0.406
Father born in Norway (%)	86.4%	85.1%	90.5%	0.791
Biological parents living together (%)	60.2%	64.2%	47.6%	0.272
Living with siblings (%)	72.2%	75.0%	63.6%	0.447
Father, full time work (%)	71.9%	71.7%	72.6%	0.908
Father, part time work (%)	1.1%	0.0%	1,5%	0.549
Mother, full time work (%)	52.2%	53.0%	45.0%	0.777
Mother, part time work (%)	18.2%	20.6%	10.0%	0.505
Father, completed education (%)				
≤High school	62.6%	54.9%	85%	0.066
College/University <4 years	20.0%	23.3%	10%	
College/University >4 years	11.3%	13.3%	5%	0.088
Mother, completed education (%)				
≤High school	58.5%	56.7%	65.0%	0.685
College/University <4 years	23.0%	25.0%	15.0%	
College/University >4 years	18.4%	17.9%	20.0%	0.386

*Note*: *p* values from a chi‐square test for categorical data, and independent samples t‐test for continuous data. The categories < or >4 years of College/University were merged for the group comparisons.

Abbreviations: BMI, body mass index; SD, standard deviation.

81 of 90 families (90%) participating in FBSFT filled out the BTPS. The therapist questionnaire was filled out for 86 of 90 families (95.5%). For three families both parent‐ and therapist questionnaire was missing, for six families only the parent questionnaire was missing, and for one family only the therapist questionnaire was missing.

Means, standard deviations and Pearson correlations between the BTPS subscales are presented in Table 3, whereas Cronbach's alphas and correlations between family‐ and therapist ratings are presented in Table 4. Internal consistency of the subscales was acceptable in general. There was a high correlation between family and therapist scores for three subscales, but not for the scale *Relationship with the therapist*.

**TABLE 3:** Means, standard deviations and pearson's correlations r between subscales of the barriers to treatment participation scale for family and therapist reports (*N* = 81)

		1	2	3	4	Mean	SD
1.	Competing stressors and obstacles	—	0.53	0.43	0.54	1.77	0.50
2.	Treatment demands	0.53	—	0.70	0.57	1.67	0.51
3.	Relevance of treatment	0.37	0.59	—	0.52	1.87	0.56
4.	Relationship with therapist	0.44	0.59	0.55	—	1.44	0.49
Mean		1.77	1.57	1.89	1.23	—	—
SD		0.46	0.44	0.53	0.39	—	—

*Note*: Family ratings are presented below the diagonal and therapist ratings are presented above the diagonal. All correlation *r*s are statistically significant at *p* < 0.001.

Abbreviation: SD, standard deviation.

**TABLE 4:** Cronbach's alphas and correlations between family‐rated and therapist‐rated barriers to treatment

Variables		R	*α* (family/therapist)
1.	Competing stressors and obstacles	0.53***	0.83/0.87
2.	Treatment demands	0.43***	0.61/0.72
3.	Relevance of treatment	0.37***	0.64/0.71
4.	Relationship with therapist	0.16	0.77/0.84

*** *p* < 0.001.

### 3.1 Parent version of BTPS, families who completed versus did not complete FBSFT

The Hotelling's *T* ^2^ test indicated differences between those who did (*n* = 65) and did not (*n* = 16) complete FBSFT on the BTPS subscales, *T* ^2^ = 16.645, *df* = 3,77, *p* = 0.002. The Box M test was not statistically significant, *F* (6,4308.9) = 1.04, *p* = 0.39, indicating that the covariance matrices were not different, and that the assumption of homogeneity is not violated.

The post‐hoc comparison of mean scores on the different subscales (Table 5) showed that families who did versus did not complete treatment differed on the subscales *Stressors and obstacles that compete with treatment* and *Perceived relevance of treatment*. Families who did not complete FBSFT scored significantly higher on *stressors and obstacles* (*M* = 2.03, *SD* = 0.53) than those who completed treatment (*M* = 1.70, *SD* = 0.42), *T* = 2.625, *p* = 0.010. Furthermore, families who did not completed FBSFT scored significantly higher on *relevance of treatment* (*M* = 2.27, *SD* = 0.48) than those who completed treatment (*M* = 1.80, *SD* = 0.50), *T* = 3.458, *p* < 0.001. The mean differences in *stressors and obstacles* (Cohen's *D* = 0.73) and *treatment relevance* (Cohen's *D* = 0.97) represent medium‐to‐large and large effect sizes, respectively.

**TABLE 5:** Differences in parent‐reported barriers to treatment between families who did and did not complete treatment

Subscale	Completed		*n*	Not completed		*n*	T	*p* ^a^	D
	M	SD		M	SD				
Competing stressors and obstacles	1.70	0.42	65	2.03	0.53	16	2.625	**0.010**	0.73
Treatment demands	1.53	0.43	65	1.73	0.46	16	1.586	0.117	0.44
Relevance of treatment	1.80	0.50	65	2.27	0.48	16	3.458	**<0.001**	0.97

*Note*: Hotelling *T* ^2^ = 16.645, with Mahalanobis *D* ^2^ = 0.42. Higher scores indicate greater presence of barriers to treatment.

Abbreviations: M, mean; SD, standard deviation.

^a^ *p*‐values in bold indicates statistically significant values after applying a Bonferroni correction (αm=0.053=0.016).

The Wilcoxon rank‐sum test that compared the groups on *Relationship with the therapist* showed that the two distributions were not statistically different at a 0.05 significance level, *Z* = 1.462, *p* = 0.144. The probability of a random case from the group that did not complete FBSFT having a higher score on *Relationship with the therapist* was not much higher than chance (*p* = 0.61).

The Wilcoxon rank‐sum test that compared families who completed versus did not complete on the number of reported critical events also showed that the two distributions were not statistically different at a 0.05 significance level, Z = 1.237, *p* = 0.216. Among families who completed treatment (*N* = 65), 66.2% reported no critical events, while 18.5% reported one, 7.7% two, 3.1% three and 4.5% four critical events. Among families who did not complete treatment (*N* = 16), 81.3% reported no critical events, while 12.5% reported one and 6.2% reported two critical events.

### 3.2 Mean ratings for family and therapist versions of the BTPS

The 10 barriers with highest mean rating for families and therapists are reported in Table 6. For both groups, the barrier *during the course of treatment I (the parent) experienced a lot of stress in my life* was the barrier with highest mean rating. Thereafter, the rank of barriers differs between families and therapists.

**TABLE 6:** The ten barriers with highest mean ratings for families and therapists

#	Subscale	Item content	Family		Therapist	
			M	Rank	M	Rank
4	CS	Scheduling of appointment times for treatment	1.77	17	1.98	**10**
6	CS	Treatment was in conflict with other activities (classes, job, friends)	2.72	**2**	2.31	**5**
7	TR	Treatment did not seem necessary	2.20	**7**	1.73	18
11	TR	Treatment was not what expected	2.30	**4**	2.07	**8**
14	CS	During the course of treatment parent experienced a lot of stress in life	2.99	**1**	3.03	**1**
20	CS	Treatment added another stressor to life	2.04	**8**	2.57	**3**
22	TD	Treatment was more work than expected	1.96	11	2.20	**6**
29	TR	Child's behaviour seems to have improved, therefore, treatment no longer seems necessary	2.58	**3**	2.93	**2**
30	TR	Treatment did not seem to be working	2.03	**9**	2.19	**7**
34	CS	Did not have time for the assigned work	2.28	**5**	2.47	**4**
39	CS	Finding a place to park at the clinic	2.26	**6**	1.79	16
42	CS	Job got in the way of coming to a session	1.99	**10**	1.99	**9**

*Note*: The items are the same for both versions, with different wording. # = item number on the questionnaire. #29, score 1 = improved, higher scores indicate greater presence of barriers to treatment. Bold value rank within top ten list.

Abbreviations: CS, competing stressors and obstacles; TD, treatment demands; TR, treatment relevance.

## 4 DISCUSSION

This study demonstrated that families who did not complete FBSFT reported significantly more barriers related to the subscales *stressors and obstacles that compete with treatment* and *perceived relevance of treatment* than families who completed treatment. No group differences were observed for the *treatment demands and issues* and *relationship with the therapist* subscales. The barrier *during the course of treatment I (the parent) experienced a lot of stress in my life* was highest ranked both by parents and therapists. To our knowledge, this is one of the first studies comparing perceived barriers for treatment participation in families who did versus did not complete an enhanced FBT for paediatric obesity.

### 4.1 Stressors and obstacles that competed with treatment

Families who did not complete FBSFT reported more perceived stressors and obstacles compared to those who completed treatment. This subscale consists of a wide range of barriers related to events interfering with the ability to attend sessions and treatment serving as, and adding to, other stressors experienced in the family.^26^ Our finding is in line with previous research, reporting high degree of family stressors as a challenge for treatment adherence.^6^, ^11^ Across all participating families in our study, the barrier *during the course of treatment I (the parent) experienced a lot of stress in my life* was the most prevalent, followed by *treatment conflicting with other activities*. Out of the 10 barriers with highest mean ratings for participating families, six were from the stressors and obstacles subscale. This finding, describing a patient group experiencing a high burden of life stressors, aligns with previous literature on families seeking paediatric obesity treatment.^27^, ^28^ The associations between family stress (including both parental perceived stress and stress across the entire home environment) and paediatric obesity are complex, and need to be further investigated to enhance our understanding of their impact on treatment engagement.^29^, ^30^ In addition, the experience of stress warrants further investigation, as families experience stress in different ways and parents' response to stress varies.^29^ The present study show that the families with the highest degree of competing stressors and obstacles were more likely to leave treatment prematurely. Stressors can be both psychological (e.g., health issues, conflicts, crisis) and logistical, and some of the logistical challenges might be easy to work around if the therapist/clinic is aware of them. In our study, the barrier *finding a place to park at the clinic* had the sixth highest mean rating among families, while for therapists it was ranked as number sixteenth. Increasing therapists' awareness of these issues can increase the likelihood of addressing them. For example, if the therapists had been more aware of this barrier, they could have helped families finding a suitable parking arrangement.

### 4.2 Perceived relevance of treatment

This subscale, which reflects the extent to which treatment was seen as relevant to the child's problem, was viewed as important, and met with the families' expectations and needs.^26^ Significantly less burden was reported among families who completed FBSFT. These data suggest that the intervention was perceived as less able to meet the expectations and needs of families who did not complete treatment. Previous studies on paediatric obesity also report treatment not meeting expectations as a barrier for participation,^3^, ^6^ and mainly it seems like this barrier is related to not achieving the desired weight loss effect.^6^ Such outcomes may reflect participants' desires for weight loss that often are accompanied by unrealistic expectations going into the intervention.^6^, ^31^ FBSFT has a modest weight loss goal with focus on long‐term healthy lifestyle changes,^19^ possibly in conflict with the expectations of some of the enrolled families, and thereby potentially increasing risk for dropout.^31^ Another issue related to perceived relevance of treatment is parents' divergent views about paediatric obesity,^11^ with some parents considering the condition as not in need of treatment. Not viewing obesity as a problem is a known barrier during admission to treatment.^6^, ^32^ In our study, the participating families actively agreed to take a more intensive treatment approach,^21^ but ambivalence concerning whether the treatment is necessary was still present in the study population: The barrier *treatment did not seem necessary* was the seventh most frequently reported barrier among families. However, the barrier *my child's behaviour seems to have improved, therefore, treatment no longer seems necessary* (score of 1 = improved) is ranked as number three. Nevertheless, the observed differences between non‐completers and completers on this subscale highlights the importance of supporting families in identifying, discussing, and managing their expectations and collaboratively establishing realistic treatment goals.^31^

### 4.3 Treatment demands and issues and relationship with the therapist

No differences between those who did and did not complete FBSFT were observed for the *treatment demands and issues* and *relationship with therapist* subscales. These findings contrast with previous studies that reported barriers related to treatment demands, especially regarding collection of research data, and dissatisfaction with treatment providers as a reason for ending treatment prematurely.^2^

The *treatment demands and issues* subscale reflects the families' concerns and complaints related to treatment participation and the extent to which the treatment was considered confusing, too long, difficult or demanding.^26^ In addition to no differences between those who did and did not complete, none of the barriers on this subscale were on the top 10 list for the total sample of participating families. This is of course encouraging, but also a bit surprising. From a clinical perspective, FBSFT is perceived as requiring a lot of work from the families (e.g., frequent sessions, monitoring behaviours, homework), which may explain why therapists rated the barrier *treatment was more work than expected* higher than families (rank 6 versus 11).

Furthermore, the *relationship with the therapist* subscale investigated alliance, bonding, liking of, perceived support from and disclosure with the therapist.^26^ Within psychotherapeutic approaches, the therapeutic alliance is a known predictor for patient outcomes.^33^, ^34^ Our study was not able to detect any group difference related to treatment completion, as the whole group of participating families had a low mean score on this subscale. However, it is very positive for the FABO study and the FBSFT intervention that participating families experienced a supportive, strong therapeutic alliance with their therapist. Stigmatization and unequal treatment within the healthcare system have previously been reported for both children and adults with obesity,^35^, ^36^, ^37^ and a lack of trust and connection with healthcare providers represent barriers for adherence in paediatric obesity treatment.^7^

### 4.4 Strengths and limitations

There are multiple strengths of the present study. The main strength of this study is the inclusion of all families that received FBSFT, both those who did and did not complete treatment. In addition, 90% of participating families filled out the BTPS. All families were informed that their therapist was not given access to their scores, reducing the risk for social‐desirability bias. Furthermore, the use of both parent and therapist versions of the BTPS is novel and made it possible to compare scores and compare perceived barriers among recipients and providers of FBSFT. The study has limitations. The BTPS is often administered by means of an interview,^8^, ^9^, ^26^ which may provide more precise answers than the questionnaire format. Furthermore, the BTPS was filled out by the parents. Inclusion of a self‐report version for the participating adolescent would have provided valuable insight into their own experienced barriers. Inclusion of qualitative interviews in addition to the use of the BTPS could also have broadened the understanding of the phenomenon. Lastly, due to the sample size, we could not differentiate between the timing of dropout (e.g., early or late), but previous research has shown that there may be meaningful difference based on the timing of dropout.^2^

### 4.5 Implications for practice

The results from this study indicate that barriers for participation should be investigated ahead of, during, and when leaving treatment. Examination at multiple time points will enable discussions of barriers and identifications of modifiable components that can be addressed as a part of treatment, and may optimize families' experience during, participation and completion of treatment for paediatric obesity.

Our finding that families with a high degree of stressors and obstacles were more likely to dropout is important to note. Offering families practical support with day‐to‐day tasks as a part of treatment may prevent dropout and improve treatment impacts for families. At Norwegian obesity clinics, these kinds of support have been offered to some families in collaboration with the child welfare service/medical social workers. Furthermore, implementing methods of service delivery that are better suited to the logistical challenges experienced by the families are of great importance. In this study, sessions were delivered during daytime clinic hours, and a potential modification would be to also facilitate evening sessions for families.

A pre‐treatment phase to discuss and manage families' expectations and collaboratively establish realistic goals may serve as a valuable addition to FBSFT and paediatric obesity treatment delivery.^31^ Unrealistic expectations related to weight effect is a major barrier to participation, while having a positive and realistic expectations is an facilitator for completion.^6^, ^38^ Other facilitators for treatment completion and overcoming perceived barriers should also be further investigated. Previous research has demonstrated that the main reason for adherence was a personalized approach by the treatment provider, and the providers effort to establish a personal connection.^6^

### 4.6 Conclusion

The results from this study indicate that families participating in family‐based behavioural social facilitation treatment for paediatric obesity are more likely to dropout, when experiencing a high burden from life stressors or when treatment is not meeting expectations and perceived needs of the family. Identifying and addressing families' treatment expectations and how they fit with intervention as well as the degree of burden from life stressors that families are experiencing may increase their participation in and completion of family‐based treatment for paediatric obesity.

## AUTHOR CONTRIBUTIONS

Hanna F. Skjåkødegård, Rachel P. K. Conlon, Denise Wilfley, Petur B. Juliusson and Yngvild S. Danielsen conceived and designed the study. Hanna F. Skjåkødegård, Ingvild Bruserud, Petur B. Juliusson and Yngvild S. Danielsen collected and scored the data. Hanna F. Skjåkødegård, Sigurd Hystad and Yngvild S. Danielsen performed statistical analyses. Hanna F. Skjåkødegård wrote the paper in consultation with Sigurd Hystad, Ingvild Bruserud, Rachel P. K. Conlon, Denise Wilfley, Bente Frisk, Mathieu Roelants, Petur B. Juliusson and Yngvild S. Danielsen. All authors discussed the results and contributed to the final manuscript.

## CONFLICT OF INTEREST

The authors declare that there is no conflict of interest.
